# Supplementary material for: Aluminum Concentration Is Associated with Tumor Mutational Burden and the Expression of Immune Response Biomarkers in Colorectal Cancers
Source: Int J Mol Sci. 2024 Dec 13;25(24):13388. doi: 10.3390/ijms252413388 (PMC11676456; doi:10.3390/ijms252413388)
Supplement: Supplementary file 1 [file ijms-25-13388-s001.zip › ijms-3315462-supplementary.pdf]

Aluminum Concentration Is Associated with Tumor Mutational Burden and the Expression of Immune Response Biomarkers in Colorectal Cancers

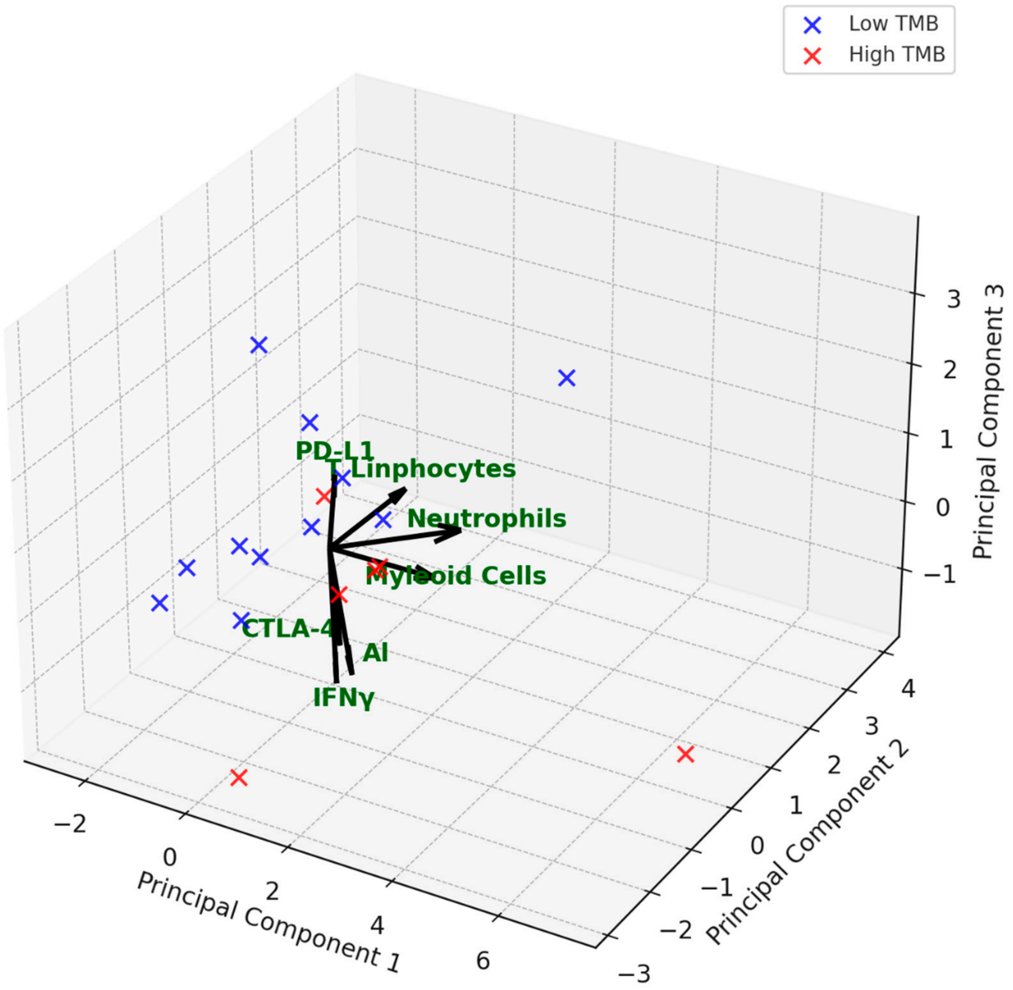

Supplementary Figure S1. 3D Biplot graph shows the main variables associated to high tumoral mutational burden (TMB); Aluminum (Al), CTLA4 and IFNγ. Variance explained by each component: principal component 1 39.44%; principal component 3 17.9%; principal component 3 13.26%.
